# User Feedback on the MSF Tele-Expertise Service After a 4-Year Pilot Trial – A Comprehensive Analysis

**DOI:** 10.3389/fpubh.2015.00257

**Published:** 2015-11-20

**Authors:** Laurent Bonnardot, Elizabeth Wootton, Joanne Liu, Olivier Steichen, Jean-Hervé Bradol, Christian Hervé, Richard Wootton

**Affiliations:** ^1^Fondation Médecins Sans Frontières, Paris, France; ^2^EA4569, Department of Medical Ethics and Legal Medicine, Paris Descartes University, Paris, France; ^3^University of Edinburgh, Edinburgh, UK; ^4^Médecins Sans Frontières, Geneva, Switzerland; ^5^McGill University, Montréal, QC, Canada; ^6^Internal Medicine Department, Tenon Hospital, AP-HP, Paris, France; ^7^Faculty of Medicine, Sorbonne Universités, University Pierre et Marie Curie, Paris, France; ^8^U1142, Laboratoire d’Informatique Médicale et d’Ingénieurie des Connaissances en e-Santé (LIMICS), INSERM, Université Paris, Paris, France; ^9^Crash, Fondation Médecins Sans Frontières, Paris, France; ^10^Norwegian Centre for Integrated Care and Telemedicine, University Hospital of North Norway, Tromsø, Norway; ^11^Faculty of Health Sciences, University of Tromsø, Tromsø, Norway

**Keywords:** telemedicine, teleconsultation, tele-expertise, humanitarian, low-resource setting

## Abstract

We surveyed all users of the Médecins Sans Frontières (MSF) tele-expertise service, approximately four years after it began operation. The survey contained 50 questions and was sent to 294 referrers and 254 specialists. There were 163 responses (response rate 30%). There were no significant differences between the responses from French and English users, so the responses were combined for subsequent analysis. Most of the responders were doctors (133 of 157 who answered that question), and most had completed field missions for MSF, i.e., both specialists and referrers. The majority stated that the system was user friendly and that they found it self-explanatory (i.e., they did not need to be shown how to use it). Almost all the referrers found that the telemedicine advice that they received was helpful, changed diagnosis and management, and/or reassured the patient. Similar feedback came from the specialists, who also felt that there was educational value for the field doctor. Although there was general satisfaction with the service, the survey identified various problems. The main concerns of the referrers were the lack of promotion of the system at headquarters’ level, and the main concerns of the specialists were the lack of feedback about patient follow-up. Nonetheless, both referrers and specialists recognized the benefits of telemedicine in improving patient management, providing education, and reducing isolation in the field.

## Introduction

In 2009, Médecins Sans Frontières (Doctors Without Borders, MSF) began a pilot trial of a store-and-forward medical tele-expertise service. “Tele-expertise” is defined in the French Public Health Code (1), as one of the five main areas of telemedicine (teleconsultation, tele-assistance, tele-monitoring, medical emergency call center, and tele-expertise). The MSF system uses electronic message exchange between field clinicians working in resource-limited settings without access to specialized medical expertise, and specialists working in relatively well-resourced settings who offer *pro bono* specialist advice to their field colleagues. In collaboration with the Swinfen Charitable Trust (2), three separate tele-expertise networks (French, English, and Spanish) were established initially and then combined into a single multilingual network in the late 2013, using web-based technology based on the Collegium Telemedicus system (3).

The MSF tele-expertise service provides specialized advice to field clinicians across a very wide range of medical and surgical specialties. At present more than 350 MSF field clinicians have access to the system, and there are 300 volunteer specialists from all over the world who provide tele-expertise; the median delay in providing the first specialist response to the referrer is less than 6 h (4).

To ensure that the allocation of resources is appropriate in health care, pilot projects require careful evaluation. In the clinical field, such an evaluation can also be considered as a necessary reflective practice, carried out in order to improve both the efficiency of the system and provider practice (5). We therefore surveyed the users of the MSF pilot tele-expertise service to obtain their feedback in terms of satisfaction and benefit, to draw conclusions about the usefulness of the service, and to identify potential areas of improvement. In a previous paper, we presented the main results of the survey (4). The present study provides the raw data and a further and more detailed analysis. In order to understand the results and put them in perspective, we compared them with the (limited) data which has been published on other, similar telemedicine networks (6, 7).

## Materials and Methods

We carried out a survey of all users with an account on the tele-expertise network in January 2014. The questions were established after conducting a literature search combined with qualitative data collection:

1. in-depth interviews with three referrers and three specialists;
2. participant observation performed by two of the authors during several field missions in MSF settings (Democratic Republic of the Congo, Central African Republic, Yemen, Somalia, Syria, and Haiti).

The survey contained 50 questions. They were closed-ended, multiple choice and scale type questions, and open-ended questions.

The survey was tested on three referrers and three specialists, in English and in French. After the pilot testing, the survey was sent separately to all referrers (French and English) and specialists (French and English) with accounts on the system, regardless of whether they were active (i.e., irrespective of whether they had logged in and submitted cases or answered them). The questions for referrers and specialists were almost identical. Versions of the survey were made available in French and English. Web-based survey software (https://www.surveymonkey.com/) was used to collect the data. Two reminders were sent by email to the users, after 1 and 3 weeks. Data collection was closed after 1 month.

Ethics permission to conduct the survey was not required because patient consent to access the data had been obtained previously, and the work was a retrospective chart review conducted by the organization’s staff in accordance with its research policies (8).

Survey responses were examined with the usual methods for quantitative analysis, while the results of the open-ended questions were processed in a qualitative way based on a systematic content analysis.

### Differences Between Language Groups

Responses were received to both the English and French versions of the survey. To examine potential differences between the two language groups, the English and French responses were compared using chi-squared tests for certain key variables:

1. Age. The respondents’ ages were recorded in five age categories. Since the age categories were ordered, a chi-squared test for trend was carried out.
2. Qualifications. The respondents’ qualifications were recorded in three categories. These were unordered categories, so Fisher’s exact test was used with *P*-values calculated according to the Freeman–Halton extension (9, 10).
3. User-friendliness of the system interface. The respondent’s opinion about the user-friendliness of the system was categorized as yes/no, so Fisher’s exact test was used.
4. Value of telemedicine. The respondent’s opinions about whether telemedicine improved patient management was recorded in three unordered categories, so Fisher’s exact test was used with *P*-values calculated according to the Freeman–Halton extension (9, 10).

### Differences Between Referrers and Specialists

Responses from referrers and specialists were compared, irrespective of language, using chi-squared tests. Where multiple ordered responses were possible, the chi-squared test for trend was employed.

## Results

### Users and Response Rate

A total of 294 referrers and 254 specialists had accounts on the MSF network. From an examination of the system database, a total of 104 referrers (35%) had submitted cases and 120 specialists (47%) had replied to queries, i.e., 224 of the 548 account holders could be considered as to be active users (41%).

**Figure 1:**
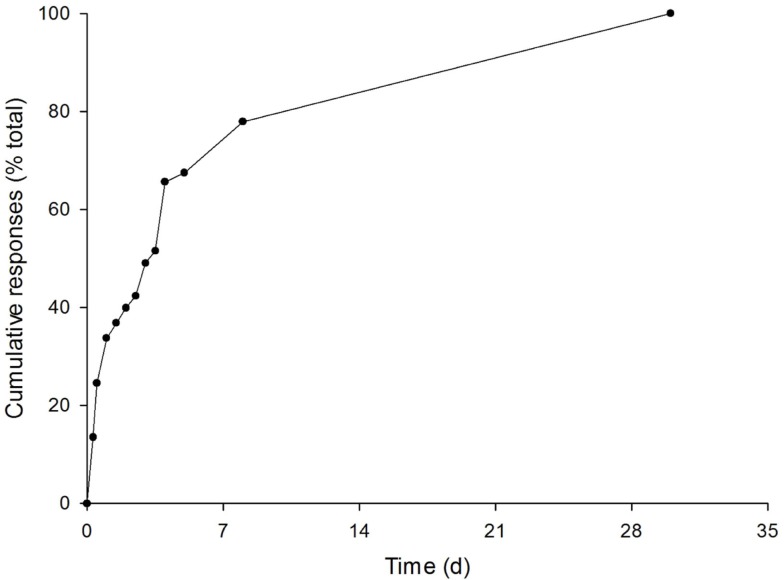
**Cumulative number of responses to the survey (*n* = 163)**.

The survey was sent to all referrers and specialists. Of the 548 people who were sent the survey, 163 (30%) responded: the group details are summarized in Table 1. The survey was completed reasonably promptly. Non-linear regression showed that during the first week, the numbers of responses received doubled every 1.8 days (*R*^2^ = 0.94). Seventy percent of the questionnaires were completed within 6 days (Figure 1).

**Table 1:** **Responses from referrers and specialists**.

	Total number in group	Number of responses in English	Number of responses in French	Total number of responses (%)
Referrers	294	52	12	64 (22)
Specialists	254	68	31	99 (39)
Total	548	120	43	163 (30)

By matching answers to some questions (such as whether they found the system easy to use and how long it took to write a referral or answer a case), the survey showed that approximately 40 of the 64 referrers who responded (62%) and 70 of 99 specialists who responded (70%) could be considered as active users. Although there were more referrers (54%) than specialists (46%) in the population sampled, specialists provided significantly more answers than referrers – 39 versus 22%. This was consistent when considering the individual item response rates.

### Differences Between Main Categories

There were no significant differences between the responses from French and English users (Table 2), so the responses were combined for subsequent analysis. The survey questions were divided into five main categories (see Table 3):

**Table 2:** **Differences between the responses from those completing the French and English versions of the questionnaire**.

	Possible responses	Referrers	Specialists
Age (years)	25–34, 35–44, 45–54, 55–64, >65	*P* = 0.14	*P* = 0.90
Qualifications	Medical doctor, nurse, and others	*P* = 0.61	*P* = 0.31
System is user friendly	Yes and no	*P* = 0.17	*P* = 0.10
Telemedicine is valuable	Yes, no, and unknown	*P* = 0.73	*P* = 0.51

*The *P*-values shown are for the tests described in the text*.

**Table 3:** **Survey results**.

Question	Response (majority response in bold with %)	Total answered	Skipped
**(A) GENERAL USER PROFILE**			
**Referrers**			
1. How old are you?	Mean 41 years	63	1
2. What is your nationality?	Spain 6, Canada 6, France 6, Belgium 4, Netherlands 3, Kenya 3, Colombia 3, 8 other countries (2 referrers each) 16, and 13 other countries (1 referrer each) 13	60	4
3. What is your qualification and/or field of expertise?	**Doctors (73%)** 46, nurses 12, and others 5	63	1
4. How many missions (MSF or other field experiences) have you undertaken?	<1 1, 1–2 14, 3–5 13, and **>5 (54%)** 33	61	3
5. How long is the total (cumulative) duration of these missions?	>5 years, <1 year 5, 1–2 years 16, 3–5 years 12, and **>5 years(46%)** 28	61	3
6. What was your job position when a system user?	**Medical team leader (44%)** 21, medical coordinator 20, regular volunteer 7, first mission 4, and others 16	48	16
**Specialists**			
1. How old are you?	Mean 47 years	99	1
2. What is your nationality?	France 20, Canada 17, Spain 10, US 8, UK 6, Netherlands 5, Argentina 4, Colombia 3, 4 other countries (2 specialists each), and 16 other countries (1 specialist each)	97	2
3. What is your qualification and/or field of expertise?	**Doctors (93%)** 87, nurses 2, and others 5	94	5
4. Where is your work location?	Public health service 28, **teaching hospital (45%)** 44, private 13, NGO 28, retired 3, and others 6	98	1
5. How many missions (MSF or other field experience) have you undertaken?	<1 33, 1–2 14, 3–5 12, and **>5 (38%)** 37	96	3
6. How long is the total (cumulative) duration of these missions?	<1 year 28, **1–2 years (37%)** 19, 3–5 years 16, and >5 years 12	75	24
7. In which year was your last mission?	1971–1980 1, 1981–1990 1, 1991–2000 2, 2001–2010 23, and **2011–2014 (52%)** 36	69	30
**(B) IT PROFILE**			
**Referrers**			
7. Are you involved in any other telemedicine project?	Yes 12 and **no (80%)** 49	61	3
8. During your mission, how many times per day do/did you usually check your emails?	<1/day 6, 1/day 8, **>2/day (58%)** 34, and continuously (e.g., smartphone) 13	59	4
9. When off mission, how many times do you check your emails during the day?	<1/day 2, 1/day 10, **>2/day (57%)** 35, and continuously (e.g., smartphone) 14	61	3
10. Do you have access to Internet at work?	None 0, not easily 10, **easily (65%)** 40, and continuously (e.g., smartphone) 11	61	3
11. How was the Internet connection quality and speed?	Very low 2, low 10, **middle (52%)** 32, and high 17	61	3
12. Are you able to send files attached to emails?	Easily whatever the attachment size 15, **easily if small attachment (59%)** 36, with difficulty 10, and impossible 0	61	3
13. What type of connection do you mainly use?	**Wifi (60%)** 36, Ethernet 14, modem 14, mobile 6, and Vsat 5	60	4
14. What is usually the duration of your Internet connection?	<2 min 3, 2–5 min 2, 6–20 min 11, and **>20 min (74%)** 45	61	3
15. What type of equipment do you have?	Mobile phone with email 18, **laptop (95%)** 58, tablet 7, and others 2	61^a^	3
16. What other networks do you use?	**Facebook (70%)** 37, Twitter 14, professional medical network 27, and others 3	53^a^	11
**Specialists**			
8. Are you involved in any other telemedicine project?	**Yes (74%)** 25 and no 73	98	1
9. How many times do you check your emails during the day?	<1/day 5, 1/day 18, >2/day 34, and **continuously (44%)** (e.g., smartphone) 42	96^a^	3
10. Do you have access to the Internet at work?	None 0, not easily 3, easily 45, and **continuously (51%)** (e.g., smartphone) 49	97	2
11. How was the Internet connection quality and speed?	Very low 1, low 2, middle 25, and **high (71%)** 68	96	3
12. Are you able to send files attached to emails?	**Easily whatever the attachment size (52%)** 51, easily if small attachment 46, with difficulty 1, and impossible 0	98	1
13. What type of connection do you mainly use?	**Wifi (66%)** 64, Ethernet 30, modem 12, and mobile 14	97	2
14. What is usually the duration of your Internet connection?	<2 min 3, 2–5 min 2, 6–20 min 11, and **>20 min (83%)** 79	95	4
15. What type of equipment do you have?	Mobile phone with email 56, **laptop (87%)** 85, tablet 32, and others 17	98	1
16. What other networks do you use?	Facebook 33, Twitter 7, **professional medical network (70%)** 46, and others 10	66	33
**(C) USE OF THE TELEMEDICINE NETWORK**			
**Referrers**			
17. Did you receive any briefing about the system prior to departure?	Yes 21 and **no (56%)** 27	48	16
18. Do you find the design user friendly?	**Yes (84%)** 36 and no 7	43	21
19. Did you find it self-explanatory (i.e., there was no need to be shown how it works)?	**Yes (58%)** 24 and no 17	41	23
20. Did you encounter any problems with the username or password?	**Never (45%)** 19, **sometimes (45%)** 19, regularly 3, and always 1	42	22
21. Did you encounter any problems of connection while using the interface?	**Never (49%)** 20, sometimes 16, regularly 5, and always 0	41	23
22. If a problem occurred, was it easy to solve?	**Yes (45%)** 20, no 5, and not applicable 19	44	20
23. If a problem occurred, was it easy to contact the system operator for support?	Yes 20, no 4, and **not applicable (47%)** 21	45	19
24. How long on average does it take to write a clinical case?	<5 min 4, 5–10 min 11, **10–20 min (32%)** 13, and >20 min 12	40	24
25. How did you write your referral?	Online 13 and **offline (and then copy and paste it in) (67%)** 26	39	25
26. Did you find it easy to send an attached file?	**Yes (55%)** 23, no 10, and not applicable 9	42	22
27. Have you ever given any information about the system to a patient before sending a case?	**Yes (70%)** 26 and no 11	37	27
28. How did you get his/her consent?	**Orally (78%)** 28, written 0, and no consent 9	36	28
29. Which useful documents do you think should be linked on the website?	MSF clinical guidelines 15, essential drugs 11, **medical report template (36%)** 30, technical advice (e.g., taking pictures and sending attachments) 26, and others 2	84 responses and 38 responders	26
37. Did you give back to the specialist any feedback about this patient?	Yes 15 and **no (55%)** 18	33	31
38. If No, was it because.(tick any that apply)	Patient lost to follow-up 5, **lack of time (30%)** 11, forgotten to update 9, feeling it was not necessary 6, worse outcome of patient died 1, and difficulties with Internet access 5	37 responses and 21 responders	43
40. In your opinion, is the patient likely to be available for follow-up in 2–4 months?	Yes 8, **no (46%)** 17, and do not know 12	37	27
**Specialists**			
17. Did you receive any briefing about the system?	**Yes (55%)** 48 and no 40	88	11
18. Do you find the design user friendly?	**Yes (77%)** 63 and no 19	82	17
19. Did you find it self-explanatory (i.e., there was no need to be shown how it works)?	**Yes (79%)** 64 and no 17	81	17
20. Did you encounter any problems with the username or password?	**Never (48%)** 38, sometimes 30, regularly 9, and always 2	79	20
21. Did you encounter any problems of connection while using the interface?	**Never (76%)** 60, sometimes 17, regularly 1, and always 1	79	20
22. If a problem occurred, was it easy to solve?	Yes 29, no 8, and **not applicable (53%)** 42	79	20
23. How long on average does it take to write your answer to a clinical case?	<5 min 5, 5–10 min 11, 10–20 min 24, and **>20 min (33%)** 32	72	27
24. How did you write your referral answer?	**Online (68%)** 50 and offline (and then copy and paste it) 25	74	25
25. Did you find it easy to send an attached file?	**Yes (48%)** 37, no 6, and not applicable 34	77	22
27. How many cases did you answer?	0–1 9, **1–5 (49%)** 38, 6–10 14, and >10 7	78	21
28. Was the information (including any images) supplied by the referrer adequate?	**Yes (66%)** 44 and no 22	66	33
29. Was information about the hospital available on the website (e.g., information about the staff and facilities)?	Absent 18, insufficient 21, **sufficient (35%)** 22, and easily accessible and complete 1	62	37
30. Was the question asked by the referrer clear?	**Yes (92%)** 59 and no 5	64	35
31. Was it difficult to find the time required to answer this case?	Yes 22 and **no (66%)** 42	64	35
37. Did you receive any follow-up information about this patient?	Yes 5 and **no (92%)** 58	63	36
**(D) OUTCOMES**			
**Referrers**			
30. Did you find the advice helpful?	**A lot (42%)** 14, moderately 13, a little 3, and not at all 3	33	31
31. If yes, did it. Please tick any that apply^a^: change your diagnosis, change your management of the patient, improve the patient’s symptoms, improve the patient’s function, and provide reassurance to you or the patient	Change your diagnosis 12, change your management of the patient 21, improve the patient’s symptoms 10, improve the patient’s function 6, and **provide reassurance to you or the patient (34%)** 25	74 responses and 31 responders	33
32. Was the answer appropriate and adapted to your field environment?	A lot 10, **moderately (61%)** 20, a little 1, and not at all 2	33	31
33. Do you think that the advice you received from the specialist improved the management of the patient?	**Yes (79%)** 26, no 2, and unknown 5	33	31
36. Was there any educational benefit to you in the reply?	No 2, a little 8, **moderately (39%)** 13, and a lot 10	33	31
42. Do you have any concerns about the telemedicine process?	**No (76%)** 28 and yes 9 (see Table 4)	37	27
43. Would you recommend using the system to colleagues?	**Yes (97%)** 33 and no 1	34	30
44. Overall, how would you rate your user satisfaction on a scale from 0 to 10?	Average 7.61	36	28
45. In which specialty do you think that the system is the most useful?^a^	**All specialties (28%)** 20, medical specialties (e.g., infectious diseases) 17, surgical specialties (e.g., orthopedics) 4, radiology 11, pediatrics 12, subspecialties (e.g., neuropediatrics) 7, and others 1	72 responses and 40 responders	24
46. In a low-resource setting, do you think that access to a specialist doctor can help the field doctor? (visual scale from 0 to 10)	Average 8.27	41	23
47. Do you think that field doctors are isolated in their practice in the field? (visual scale from 0 to 10)	Average 6.76	41	23
48. Do you think that this system of assistance can help the referring doctors? (four-point scale from not at all to a lot)	(a) Overall: average 3.65	37	27
	(b) To feel less isolated: average 3.67	35	29
**Specialists**			
34. Do you think the advice you provided improved the management of the patient?	**Yes (43%)** 30, no 2, and unknown 35	69	30
35. Do you think that there is any educational benefit for the field doctors when receiving the reply?	No 1, a little 9, **medium (52%)** 40, and a lot 26	76	23
36. Did the consultation have any value for you personally?	No 11 and yes 56 [mainly clinical 11, mainly educational 7, **both clinical and educational (42%)** 30, and others 12]	71 responses and 67 responders	32
39. Do you have any concerns about the telemedicine process?	**No (78%)** 59 and yes 17 (see Table 5)	76	23
41. Generally speaking, how would you rate your satisfaction of the system on a scale from 0 to 10?	Average 6.63	73	26
43. In a low-resource setting, do you think that access to a specialist doctor can help the field doctor? (visual scale from 0 to 10)	Average 8.04	82	17
44. Do you think that field doctors are isolated in their practice in the field? (visual scale from 0 to 10)	Average 7.21	81	18
45. Do you think that this system of assistance can help the referring doctors? (six-point scale from Not at all to A lot)	(a) Overall: average 3.63	71	28
	(b) To feel less isolated: average 3.65	75	24
**(E) OTHERS**			
**Referrers**			
34. In your opinion, what is the acceptable amount of time for receiving the expert’s answer?	<6 h 4, 6–12 h 8, **12–24 h (41%)** 16, 24–48 h 9, and 48–72 h 2	39	25
35. In your opinion, what is the desirable amount of time for receiving the expert’s answer?	<6 h 15, **6–12 h (39%)** 15, 12–24 h 7, 24–48 h 1, and 48–72 h 0	38	26
39. Do you think that feedback about the patient is?	Optional 5, **desirable (43%)** 16, necessary 11, and mandatory 5	37	27
41. In your opinion, when would it be relevant to give follow-up information? (e.g. providing a progress report)	**After 1 week (53%)** 20, after 2 weeks 9, after 1 month 7, after 3 months 2, and after 6 months 0	38	28
49. Please add any other comments about the service in general, or any suggestions for improvement	See Table 4	16	48
**Specialists**			
26. Which useful documents do you think should be linked on the website?^a^	**MSF clinical guidelines (28%)** 54, essential drugs 43, medical report template 34, technical advice (taking pictures and sending attachments) 44, and others 18	193 responses and 77 responders	22
32. In your opinion, what is the acceptable amount of time in which to provide an answer?	<6 h 5, 6–12 h 10, **12–24 h (42%)** 33, 24–48 h 24, and 48–72 h 6	78	21
33. In your opinion, what is the desirable amount of time in which to provide an answer?	<6 h 23, **6–12 h (26%)** 20, 12–24 h 23, 24–48 h 12, and 48–72 h 0	78	21
38. Do you think that feedback about the patient is?	Optional 1, **desirable (52%)** 35, necessary 19, and mandatory 12	67	32
40. Are you happy to provide consultations for another non-MSF network in the future?	**Yes (81%)** 63 and no 15	78	21
42. In which specialty do you think that the system is the most useful?^a^	**All specialties (45%)** 54, medical specialties (e.g., infectious diseases) 18, surgical specialties (e.g., orthopedics) 7, radiology 17, pediatrics 8, subspecialties (e.g., neuropediatrics) 10, and others 7	121 responses and 79 responders	20
46. What is your main motivation to act as a volunteer for the system?^a^	**Taking part in MSF action (30%)** 56, keeping in touch with MSF 17, interest in telemedicine 23, treating patients from low-resource settings 53, taking part in the field doctors’ training 27, and others 7	183 responses and 81 responders	18
47. Do you think that volunteering is the right status for the expert?	**Yes (94%)** 75 and no 5	80	19
48. How many cases in routine do you think you could reasonably answer?	1/day 8, 3/week 24, **1/week (36%)** 29, 1/fortnight 9, 1/month 9, and others 2	81	18
49. Do you think that the expert should receive payment?	Yes 4 and **no (95%)** 76	80	19
50. Please add any other comments about the service in general or any suggestions for improvement	See Table 5	37	62

*^a^Respondents could select more than one choice*.

#### General User Profile (Personal, Professional)

The average age of the specialists was significantly higher than that of the referrers (*P* < 0.01). The responders were multinational, predominantly European. Most responders were medical doctors, and the majority had previously completed one or more MSF field missions (including the specialists). Further details are provided in Table 3A.

#### IT Profile (Telemedicine Experience, Habits)

Most responders had access to email, usually via a laptop computer or a mobile phone. Three-quarters of the responders had used social media, such as Facebook and Twitter, for general communications, with a small difference (*P* = 0.04) between specialists (67%) and referrers (83%). Further details are provided in Table 3B.

#### Use of Telemedicine (Interface/Platform and Interaction)

Only about half of the responders (44% of the referrers and 55% of the specialists) had received a prior briefing about telemedicine. However, the majority (more than three-quarters) stated that the system was user friendly, and they found it self-explanatory (i.e., they did not need to be shown how to use it). The average time reported by referrers for writing a case was 14.7 min, and the average time reported by specialists for replying was 17.4 min.

Half of the referrers (51%) reported having connection problems, either sometimes or always, compared to 24% of specialist; this difference was significant (*P* < 0.01). Less than half of the referrers (45%) stated that they had provided feedback about the patient for the specialist; however, 92% of the specialists reported that they had not received any feedback about the patient. Further details are provided in Table 3C.

#### Outcomes (Satisfaction, Usefulness)

The majority of referrers (91%) stated that they found the advice received via telemedicine to be useful. The most common reasons for this were in providing reassurance for the referrer or patient, in changing the management of the patient and in changing the diagnosis. The majority of referrers (79%) felt that advice from the specialist improved their management of the patient. The referrers’ mean score for their satisfaction with telemedicine was 7.6 (on a scale from 1 = very unsatisfied to 10 = very satisfied).

The majority of specialists who provided an opinion (94%) thought that their advice had improved the management of the patient. The majority of specialists (99%) felt that there was educational benefit for the field doctor. The specialists’ mean score for their satisfaction with telemedicine was 6.6 (on a scale from 1 = very unsatisfied to 10 = very satisfied). Further details are provided in Table 3D.

#### Other (Referrals, Opinions)

Both referrers and specialists indicated the time in which it was desirable to receive (or provide) the response, by choosing from a series of time intervals. Using the midpoint of these time intervals to summarize the frequency distributions showed that the referrers felt that it was desirable to receive the specialist’s response within 9 h, and that the specialists thought that it was desirable to provide a response within 14 h, a slightly longer delay. Similarly, almost all responders felt that receiving feedback (specialists) or providing feedback (referrers) was necessary or obligatory; only 14% of referrers and 1% of specialists felt that it was optional. Further details are provided in Table 3E.

### Free-Text Comments Analysis

The comments made by referrers and specialists in response to the open-ended questions are summarized in Tables 4 and 5, respectively. Both referrers and specialists used adjectives such as “excellent” and phrases expressing their gratitude that the system had been set up. It is worth noting that these comments emphasized the users’ involvement in the pilot and reflected their desire to improve the system where appropriate.

**Table 4:** **Comments from referrers in response to open-ended questions (numbers 42 and 49)**.

	Number of comments
Lack of headquarters’ support in using the system	5
Satisfaction (e.g., “excellent,” “congratulations,” “thank you,” “well done, ” “outstanding,” “wonderful,” and “very good”)	4
Lack of promotion of the system (internally and externally)	4
Reduced isolation of field doctors (the more remote is the setting, the more telemedicine helps)	2
Briefing to field staff should be improved (“part of the package” and “keep fighting for promoting the service”)	2
Platform “not interactive enough”: proposal to use other technology (e.g., video and SMS)	2
Difficulties in getting access to the websites	2
Specialist advice:	
“needs to be more field specific”	1
“need to make specialist well aware of field limitations”	1
“providing solutions for specialized management if not available in the field”	1
issue of delay in getting specialist reply	1
Field observation that “there is low use of the system”	1
Not all specialties covered (e.g., multidrug resistant tuberculosis)	1
Reluctance to use the system by expatriate doctors (fearing that specialized treatment recommended by specialist would be not available)	1
Creating a “link to telemedicine” in MSF clinical guidelines	1
Fear of bypassing the headquarters, medical referent if using the system	1
Decision to give access to the system only to medical coordinator (and not bedside doctors) since field medical staff should not “be service dependant” (no need of the system to take medical decision)	1

**Table 5:** **Comments from specialists in response to open-ended questions (numbers 39 and 50)**.

	Number of comments
Lack of feedback about patient follow-up	9
No case received (“frustration” and “disappointment” leading to loss of motivation/disengagement)	7
Satisfaction (e.g., “congratulations,” “excellent,” and “merci”)	3
Importance of field experience for giving a well-adapted answer (feeling of lack of experience)	2
Reasons of difficulties in giving their opinion	
lack of time	1
difficulties in giving advice without performing their own clinical/physical examination	1
lack of knowledge about local diseases and hospital setting (facilities, investigations, and drugs available)	1
Negative points about the referral received	
poor quality of images	1
improving the quality of the case reports	1
system not well adapted for emergency case	1
Negative points about the platform design	
design “unpersonal, cold”	1
needs “to be polished”	1
password forgotten: suggestion that system sends systematically a login reminder after 6 months without logging in	1
Difficulties in getting access to the websites	1
Service provides “moral (psychological?) support” and “field doctor reassurance”	1
Long-term benefit: “telemedicine can be the future” especially when finishing up an MSF project (to give assistance to local staff after the project has closed)	1
Specialist happy to volunteer for MSF, but financial incentives should be considered for those who are sent cases very frequently	1
Technical issues	
setting up a video link such as Skype, Facetime (comment from an ophthalmologist)	1
possibility of using SMS for some communications	1

The main comments from referrers focused on the lack of support, promotion, and predeparture briefing at headquarters’ level, whereas the principal concern expressed by the specialists was the lack of feedback about patient follow-up. There were some negative comments made by referrers concerning the characteristics of the expert advice received (delayed, not field specific, etc.) and the platform itself (not enough interactive communication). Such remarks may explain the reluctance of some potential users to use the system (e.g., fear of not being able to implement the expert advice received).

Anticipating a possible loss of motivation, specialists expressed their frustration in not receiving cases, which can also be seen as another consequence of the lack of promotion and the low use of the system. However, the specialists provided constructive comments about the system. One pointed out the long-term benefit of such a network, which can continue at a health care facility even after MSF support in the field has ended.

## Discussion

We conducted a comprehensive survey of the users of the MSF tele-expertise service during its first four years of operation. Our previous paper (4) summarized the main results, and the present paper provides a detailed description and analysis. There was high satisfaction with the service, among both referrers and specialists. All users recognized the benefits of providing access to specialist advice in low-resource settings where there is usually no alternative way of obtaining specialist expertise.

Although there was general satisfaction with the service, the survey identified some specific problems. For example, some referrers reported poor connectivity in the field, which affected their ability to communicate by email and to upload files via the telemedicine network. This problem is amenable to technical solution. However, the main concerns – raised by both referrers and specialists – were the lack of promotion of the system at headquarters’ level, and the lack of feedback about patient follow-up. These problems are less easy to address.

Another issue uncovered during our field participant observations was the use of uncontrolled social media for communicating confidential information in remote health care. The survey confirmed that most referrers and specialists used Facebook and/or Twitter for general communications, and personal communications from certain users confirmed that some MSF staff also used these media for obtaining second opinions about medical cases. For example, a Facebook group was created to provide access to a videolink and obtain feedback from expert friends. In contrast, the MSF tele-expertise service represents a safe and secure method of facilitating such consultations at a distance, and this aspect has been an important factor in convincing some reluctant headquarters’ stakeholders to adopt the system.

### Limitations of the Study

The study had certain limitations. For example, some of the information solicited in the survey was based on the opinions of the responders, which may itself introduce bias. Despite the methodological weakness of seeking personal opinions, it is important to conduct such surveys in order to identify areas for service improvement.

About two-thirds of the responses were from active users. The results therefore provide a view from users with practical experience of the tele-expertise service. This was supplemented by the views of the inactive users, i.e., people who were registered account holders but at the time of the survey were still awaiting an opportunity to submit a case or provide an answer. Unfortunately, the detailed characteristics of the 385 non-responders are unknown, which represents a potential source of bias. This study limitation was a consequence of the strict anonymization, which made it impossible to link the answers in the survey to individual user profiles in the system database. This in turn prevented the follow-up of the non-responders, so that we were unable to hypothesize about their reasons for non-response. One way to improve the design of a future study would be send different surveys to users who had previously been identified as active or inactive, and perhaps to differentiate the latter into those who had never logged in and those who had yet to submit or answer a case.

Another weakness of the study was the low response rate (30%). In principle, this might have been improved by extending the period for which the survey was open, but the trajectory of the responses received (Figure 1) suggests that 99% of responses were received within 2 weeks, so that the cutoff at 30 days was, if anything, conservative. Nonetheless the response rate was lower than those observed in previous surveys of doctors in industrialized countries, which have been up to twice as high. On the other hand, a survey of the users of a telemedicine network in a low-resource setting had a response rate of 19% (7).

### User Feedback

It is clear that the MSF tele-expertise service was easy to use and provided clinically useful diagnostic and management advice to clinicians in the field. Most referrers reported that the advice received via the service improved their management of the patient (30/33). Similarly, the majority of specialists who provided an answer felt that their advice had improved the management of the patient (30/32). However, a similar number of specialists did not know about the value of their advice – presumably these were specialists who had not yet answered a case. The lack of feedback about patient follow-up was the main concern expressed by the specialist. This feedback can be considered as an implied contract and is crucial in keeping our experts motivated. Well-motivated experts answer promptly, and the feedback allows them to improve and fine-tune their advice. For this purpose, the system now sends progress reports automatically (from the referrer to the specialist) that includes follow-up data.

The specialists were asked “Was the information (including any images) supplied by the referrer adequate?” Of those who responded, 33% said no. Poor quality information submitted in a referral is a common problem in store-and-forward telemedicine networks generally, and in particular it is very difficult to ensure that good quality images are supplied (11, 12). Methods for improving the quality of the information in referrals include user education and structured referral templates.

### Specialist Workforce: Cornerstone of the System

Comments made by the volunteer specialists suggested that they were highly motivated, demonstrating a high level of user participation, despite the questionnaire being rather long (50 questions). Their comments suggest that being able to show solidarity with their colleagues in the field, together with the positive image of MSF worldwide, were important factors in recruiting and keeping them motivated. Indeed, by virtue of their commitment, they played a key role in the development and the implementation of the system. All the experts who were asked to join the MSF network accepted the invitation to do so, and after four years only one specialist decided to quit because of pressure of other work. It is clear that their support and the quality of their service were vital for the system development. Their answers and free-text comments demonstrate that they see the service as more than just a way of providing a second opinion. They are fully aware that they are also providing education, support, reassurance, and reducing the isolation of the field staff.

However, in their survey responses, some of them expressed frustration about not receiving enough cases. Part of the art in managing a telemedicine network is to maintain a large enough pool of volunteer specialists to be able to answer the range of queries occurring on the network, but to keep individual specialist workloads at a reasonable level. Too many cases are likely to lead to “consultant fatigue,” whereas too few cases may lead to specialists abandoning the network. It is worth noting that the majority of specialists considered that their status (i.e., acting as unpaid volunteers) was appropriate. In terms of desirable workload, three-quarters of them mentioned that they could routinely answer several cases per week.

### Barriers to Service Adoption

Both the analysis of the free comments and the large number of inactive users among the account holders emphasize that the successful implementation of telemedicine is fraught with difficulties, including technical, cultural, legal, financial, organizational, and political barriers (13). It is clear from the literature on technology adoption that one reason for the reluctance to adopt telemedicine is that it is sometimes perceived as a threat (14) with the risk of managers losing control. In contrast, it should more properly be viewed as a complementary tool, and people should be invited to take full advantage of it, even if this necessitates changes in working practices.

The high proportion of inactive users among those registered to use the service (59%) could have several explanations, such as the rapid turnover of field staff or specialists still waiting to receive a case. In our experience, it is not unusual in store-and-forward telemedicine networks to have a substantial proportion of inactive users.

Studies of technology adoption show that there are three main reasons for under-use (15). The results of the survey suggest that service use may have been reduced for two of these reasons:

1. Lack of knowledge about the existence of the service. This seems plausible because half of potential users were not briefed before going to the field, and many users mentioned poor communication or lack of promotion of the system.
2. Lack of interest in the service by potential users. This also seems plausible because the field doctors often have very high workloads, so that access to specialized advice for non-critical cases may be considered as a luxury. In addition, the turnover of staff is high: field missions commonly last only a few months.
3. Non-delivery when the service is requested. This seems unlikely because published performance indicators show that in fact the service is delivered rapidly and at high quality (4).

### Comparison with Other Networks

A recent review identified seven store-and-forward telemedicine networks which were reasonably well-established (i.e., had been in operation for periods of five years or more) and which delivered teleconsultations to health care staff in low-resource settings (16, 17). Some of these networks have conducted user surveys, the results of which can be used as a comparator for the present study. In particular, previous surveys have asked referrers to identify the benefits of telemedicine (2, 6, 7). The results are summarized in Table 6. It is clear that referrers find these telemedicine services helpful in managing patients and in other ways, e.g., providing reassurance for patient and doctor. Nonetheless, it must be recognized that this represents low-level evidence and formal studies of the clinical and cost-effectiveness of telemedicine in low-resource settings are still awaited.

**Table 6:** **Benefits identified by referrers**.

	No of responses	%
**Patterson 2013 (67 replies)**		
Happy to use the service again	59	88
Advice was helpful	58	87
Educational benefits	50	75
Cost savings	31	46
**Zolfo 2011 (73 replies)**		
Helpful in establishing a diagnosis	30	41
Educational benefits	20	27
Literature collection	10	14
Reassurance to physician	9	12
Others (e.g., avoided patient transfer)	4	5
**Wootton 2004 (106 replies)**		
Positive opinion of the service	106	100
Happy to use the service again	106	100
Advice was helpful	99	93
Advice changed or confirmed patient management	69	65
**Present study (64 replies)**		
Educational benefits	31	93
Advice was helpful	30	91
Reassurance to physician or patient	25	81
Advice improved patient management	26	79

## Conclusion

The MSF tele-expertise service is highly regarded by field users, and the majority of specialists are satisfied with it. All users recognize the benefits of providing access to specialist advice in low-resource settings, where there is usually no other way of obtaining specialist expertise.

Both referrers and specialists recognize the benefits of telemedicine in terms of better patient management, the provision of education, and the reduction of isolation in the field. Although access to specialist advice might not be considered as a priority in low-resource settings, it is clear from the user feedback that the MSF tele-expertise service provides an answer to a real need. Much of the success of the service rests on the efforts of the volunteer specialists, who not only provide the benefits of their special expertise, but demonstrate solidarity toward their colleagues in the field in supporting their education, reducing their sense of isolation, and improving the management of their patients.

Although there was general satisfaction with the service, the survey identified some specific problems. Some of these could be solved relatively easily, such as poor connectivity in certain locations, while others, such as the lack of feedback on patients, are more difficult. Perhaps the main challenge for building a sustainable service lies in the political dimension, since improving the adoption of the system requires a strong organizational commitment. The evidence base for the tele-expertise service now seems to be irrefutable, so it is to be hoped that further diffusion will occur more rapidly.

## Conflict of Interest Statement

The authors declare that the research was conducted in the absence of any commercial or financial relationships that could be construed as a potential conflict of interest.
